# Diagnostic and therapeutic errors in trigeminal autonomic cephalalgias and hemicrania continua: a systematic review

**DOI:** 10.1186/1129-2377-14-14

**Published:** 2013-02-18

**Authors:** Michele Viana, Cristina Tassorelli, Marta Allena, Giuseppe Nappi, Ottar Sjaastad, Fabio Antonaci

**Affiliations:** 1 Headache Science Center-C. Mondino National Institute of Neurology Foundation, IRCCS, Via Mondino 2, Pavia 27100, Italy; 2 Department of Brain and Behaviour, University of Pavia, Pavia, Italy; 3 Department of Neurology, St. Olavs Hospital, Trondheim University Hospitals (NTNU), Trondheim, Norway

**Keywords:** Cluster headache, Paroxysmal hemicrania, SUNCT, Trigeminal autonomic cephalalgias, Hemicrania continua, Error, Pitfall, Misdiagnosis, Mismanagement

## Abstract

Trigeminal autonomic cephalalgias (TACs) and hemicrania continua (HC) are relatively rare but clinically rather well-defined primary headaches. Despite the existence of clear-cut diagnostic criteria (The International Classification of Headache Disorders, 2^nd^ edition - ICHD-II) and several therapeutic guidelines, errors in workup and treatment of these conditions are frequent in clinical practice. We set out to review all available published data on mismanagement of TACs and HC patients in order to understand and avoid its causes. The search strategy identified 22 published studies. The most frequent errors described in the management of patients with TACs and HC are: referral to wrong type of specialist, diagnostic delay, misdiagnosis, and the use of treatments without overt indication. Migraine with and without aura, trigeminal neuralgia, sinus infection, dental pain and temporomandibular dysfunction are the disorders most frequently overdiagnosed. Even when the clinical picture is clear-cut, TACs and HC are frequently not recognized and/or mistaken for other disorders, not only by general physicians, dentists and ENT surgeons, but also by neurologists and headache specialists. This seems to be due to limited knowledge of the specific characteristics and variants of these disorders, and it results in the unnecessary prescription of ineffective and sometimes invasive treatments which may have negative consequences for patients. Greater knowledge of and education about these disorders, among both primary care physicians and headache specialists, might contribute to improving the quality of life of TACs and HC patients.

## Introduction

The trigeminal autonomic cephalalgias (TACs) are a group of primary headache disorders that includes cluster headache (CH), paroxysmal hemicrania (PH), and short-lasting unilateral neuralgiform headache attacks with conjunctival injection and tearing/cranial autonomic features (SUNCT). Hemicrania continua (HC) is a continuous unilateral headache form that, like PH, is indomethacin-responsive. HC is included in group 4 of The International Classification of Headache Disorders, second edition (ICHD-II) [1]. However, this categorization is still debated and HC is often included with the TACs [2,3]. Moreover, some authors suggest that the two indomethacin–sensitive headaches should be in one group [4]. Compared with other primary headaches, the TACs have stereotypic features that, since they are defined in the ICHD-II diagnostic criteria [1], should, in principle, make them easily recognizable: short-lasting duration, unilateral pain location, and ipsilateral cranial autonomic symptoms (CAS). Up-to-date international therapeutic guidelines for these disorders [5] are also available. Despite these facts, diagnostic and therapeutic errors are frequently reported in the literature [6].

The aim of this study was to review all published data, available to us, on mismanagement of TACs and HC, in order to understand its causes and help improve the management of these patients. These findings have been reported in preliminary form (3^rd^ European Headache and Migraine Trust International Congress, London, September 2012).

## Review

We performed a systematic literature search for original articles reporting errors in the diagnosis, therapy or management of TACs and HC. We also looked for review articles to enrich the discussion. In addition, we considered cases of mismanagement that we have observed in our daily practice.

### Literature search

A PubMed database search was performed up to 25 September 2012, using the following “combination of terms:” (“cluster headache” OR “paroxysmal hemicrania” OR SUNCT OR “short-lasting unilateral neuralgiform headache attacks with conjunctival injection and tearing” OR SUNA OR “short-lasting unilateral neuralgiform headache attacks with cranial autonomic features” OR “hemicrania continua” OR TAC OR “trigeminal autonomic cephalalgias”) AND (error OR pitfall OR misconception OR delay OR “mis-management” OR mismanagement OR undertreatment OR undertreated OR misdiagnosis OR misdiagnosed OR underdiagnosed)”. Only articles in English were considered. We also considered articles from the reference lists of the studies found to be relevant, as well as literature known, by the authors, to be relevant.

### Data extraction

Two investigators (M.V and F.A.) separately examined the abstracts of all the articles identified in the literature search. Whenever the article title or abstract suggested that the publication might contain relevant data, the entire manuscript was examined. The following relevant data were extracted from the accepted articles: publication information (authors, years), type of study (case report/series, clinic-based study, population study), sample (number of patients), clinical data (final diagnosis, previous wrong diagnoses and related treatments, number and type of physicians consulted and time to correct diagnosis, incorrect treatments after correct diagnosis). Agreement for data extraction was good. There were only two cases of disagreements that were resolved by consensus.

### Results

**Figure 1:**
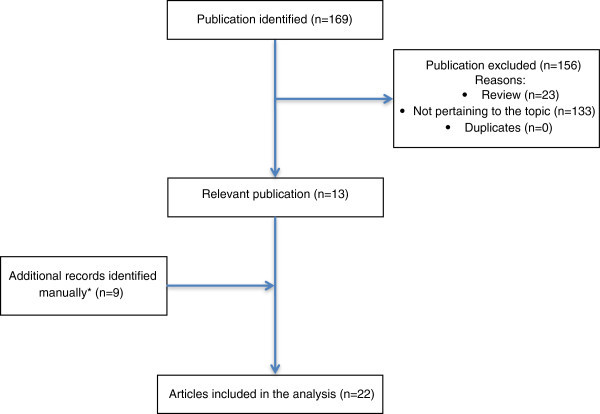
Flow-diagram of the review process. * by checking the references of relevant papers and reviews as well as literature that was known to be relevant by the authors.

The search strategy identified 169 published articles. Of these 169 papers, 13 [6-18] were relevant, while 156 did not meet the criteria (Figure 1). An additional 9 studies [16,19-25] were identified by checking the references of relevant papers and reviews, as well as literature that was known to be relevant by the authors. Finally relevant articles considered for a full text evaluation were 22. All of these 22 articles were included in the analysis. The data on errors in the diagnosis and treatment of TACs or HC extracted from the case reports/series and clinical/population studies considered in this review are summarized in Tables 1, 2, 3 and 4. The cumulative number of patients was 2614 (2593 of them stemmed from the articles found with the search method). Patients with CH were found to be by far the largest category of mismanaged patients reported in the literature (97,3% of the whole population reported in these studies). Six major studies, conducted in clinical or general population settings [6,8,13,17,18,26], investigated diagnostic and/or therapeutic errors in CH patients. A study, by Eross et al., identified one patient with CH and one with HC in a series of 100 subjects who believed they had sinus headache [11] while Sjaastad & Bakketeig interviewed 1838 inhabitans (from 18 to 65-year-old) of the Vågå commune in the mountainous area of southern Norway. Seven CH patients were observed, 6 of whom were un-aware of the diagnosis [19]. Apart from one clinical series of 33 CH patients [9] and two made up of 25 and 22 HC patients, respectively [10,15], the remaining articles were reports of single cases or small numbers of patients with TACs or HC misdiagnosed as other conditions. Data on non-optimal treatment prescribed, even after a correct diagnosis had been established, was available only for CH. This is probably explained by the fact that PH and HC are, by definition, indomethacin-responsive headaches, while SUNCT is a very rare syndrome with an extremely low number of reported cases.

**Table 1:** Data extracted from case report/series and clinical/population studies dealing with diagnostic/therapeutic errors in CH

**Authors**	**N. of pts**	**Sample**	**Methods of data acquisition**	**Diagnostic delay (means)**	**Misdiagnoses**	**Treatment before diagnosis of CH**	**Number/ type of physicians consulted prior to correct diagnosis**	**Wrong treatment after correct diagnosis**
Van Alboom et al., 2009 [6]	85	Clinic-based series	90-item questionnaire	44.4 mths	Migraine (45%), sinusitis (23%), tooth/jaw problems (23%), TTH (16%), TN (16%), ophthalmological problems (10%), neck problems (7%), nose problems (5%)	31% of pts had invasive therapy prior to CH diagnosis, including dental procedures (21%) and sinus surgery (10%)	≥3 (in 52% pts)	Propranolol (12%), amitriptyline (9%), carbamazepine (12%)
Eross et al. 2007 [11]	1	General population study (SAMS)	Direct interview	NR	Sinus headache	NR	self-diagnosed	NR
Jensen et al. 2007 [26]	85	Clinic-based series§	Semistructured telephone interview	8 yrs (range 0–35) for ECH and 9 yrs (range 0–39) for CCH	NR	Non-medical treatment was received by 58% (49/85) of the cluster patients	NR. 44.7% (38/85) of the CH pts had previously been admitted to hospital due to CH	NR
Schurks et al. 2006 [17]	246	Clinic- and non-clinic- based	Direct interview (telephone or face-to-face) or standardized mailed questionnaire	NR	NR	NR	NR	25% of patients used non-first-choice medication (such as opioids)
Bahra and Goadsby 2004 [8]	230	Non-clinic-based (76%) and clinic-based (24%)	Direct interview (telephone or face- to-face)	2.6 yrs (1990s) to 22.3 yrs (1960s)	NR	52% of pts who had been seen by a dentist or ENT surgeon had an invasive procedure	Mean 3 GPs. 2/3 of the pts seen by another specialist: dentist (45%), ENT (27%), optician (43%), opht (15%), others (7%)	Beta-blocker (43%), pizotifen (32%), TCAs (32%); alternative therapy (including acupuncture in 40%, herbal treatment in 31%, chiropractic treatment in 23%, homeopathy in 18%)
Van Vliet et al. 2003 [18]	1163	Nationwide study clinic- and non-clinic- based population	Questionnaire	3 yrs (range 1 wk–48 yrs)	Sinusitis (21%), migraine (17%), dental-related pain (11%)	Tooth extraction (16%) and ENT operation (12%)	Dentists (34%), ENT specialists (33%), and alternative therapists (33%)	NR
Sjastaad & Bakketeig, 2003 [19]	7	General population study (Vågå study) on headache epidemiology	Direct interview plus physical and neurological examination	11 yrs (range <1 – 28)	NR (5 out of 7 pts had never consulted a physician)	NR (5 out of 7 pts had never consulted a physician)	5 out of 7 pts had never consulted a physician	NR
Klapper et al. 2000 [13]	693	Internet-based survey	Internet questionnaire	6.6 yrs	3.9 (average number of incorrect diagnoses before CH) NOS	5% had surgery (mostly sinus or deviated septum surgery), other pts were prescribed with sinus medications	4.3 (3.3 gave an incorrect diagnosis)	Propranolol (27.2%) amitriptyline (16.4%), cyproheptadine (2.3%)
Hoffert 1995 [12]	1	Case report	Case report	5 yrs	Dental pain	Extractions of all the teeth	Dentist	NR
Bittar and Graff-Radford 1992 [9]	33	Clinic-based series	Review of clinical chart	8 yrs (mean duration of pain)	NR	42% of pts received inappropriate dental treatment which was often irreversible, almost all pts received different medications (NSAIDs, opiates, AEDs, TCAs)	Consultant seen before: 72% neurologist, 42% dentist, 27% internist, 12% ENT, 9% allergist	NR

CH: cluster headache; TTH: tension-type headache; TN: trigeminal neuralgia; wk: week; mths: months; yrs: years; SAMS: The Sinus, Allergy and Migraine Study; ECH: episodic cluster headache; CCH: chronic cluster headache; NSAIDs: non-steroidal antiinflammatory drugs; AEDs: anti-epileptic drugs; TCAs: tricyclic antidepressants. NOS: not otherwise specified; NR: not reported; opht: ophthalmologist. § 100 randomly chosen patients with the initial diagnosis of cluster headache seen at the Department of Neurology, Glostrup Hospital and the Danish Headache Centre between October 1998 and September 2003.

**Table 2:** Data extracted from case reports dealing with diagnostic/therapeutic errors in PH

**Authors**	**N. of pts**	**Sample**	**Diagnostic delay**	**Misdiagnoses**	**Treatment received before diagnosis of PH**	**Number/type of physicians consulted prior to correct diagnosis**
Alonso and Nixdorf 2006 [20]	1	Case report	NR	TMD	Splint therapy and bite adjustments	NR
Sarlani er al 2003 [16]	1	Case report	2 yrs	TN and sinusitis	Maxillary sinus surgery, carbamazepine and prednisone, paracetamol	NR
Benoliel and Sharav 1998 [22]	7	Case reports	10 mths (range 1–30)	Pain of dental origin (4), TMD (1), CH (1) *	2 pts had irreversible treatments (1 extraction, 1 RCT), and 1 pt received antibiotics	Mostly at least one dental practitioner
Moncada and Graff-Radford 1995 [25]	1§	Case report	12 yrs	TMD	Complete mouth reconstruction then recommendation to have condyloplasty	3 neurologists, 1 dentist, 1 oral surgeon
Delcanho and Graff-Radford 1993 [24]	2	Case report	Case 1: NR; Case 2: 3 yrs	Case 1: dental pain, migraine; Case 2: TN, TMD	Case 1: RCT, migraine prophylactic medications; Case 2: phenytoin 100 mg t.i.d.	Case 1: numerous physicians including dentist, neurologist, internal medicine specialist; Case 2: 2 dentists, 1 GP, 1 ENT specialist

PH: paroxysmal hemicrania; TMD: temporomandibular disorder; TN: trigeminal neuralgia; CH: cluster headache; mths: months; yrs: years; NR: not reported; RCT: root canal therapy; * in one patient no previous diagnosis were reported; § together with another 7 indomethacin-responsive headache patients with orofacial pain as the presenting symptom, 2 of whom were chronic paroxysmal hemicrania cases already included in a previous article [24].

**Table 3:** Data extracted from case reports dealing with diagnostic/therapeutic errors in SUNCT

**Authors**	**N. of pts**	**Sample**	**Diagnostic delay**	**Misdiagnoses**	**Treatment received before diagnosis of SUNCT**	**Number/type of physicians consulted prior to correct diagnosis**
Alore et al. 2006 [7]	1	Case report	9 yrs	TN, CH, atypical migraine	carbamazepine, phenytoin, propranolol, indomethacin and lithium	NR
Benoliel and Sharav 1998 [27]	1	Case report	2 yrs	TN	carbamazepine, baclofen, and amitriptyline	Neurologist and other physicians (NOS)

SUNCT: short-lasting unilateral neuralgiform headache attacks with conjunctival injection and tearing; TN: trigeminal neuralgia; CH: cluster headache; yrs: years; NR: not reported; NOS: not otherwise specified.

**Table 4:** Data extracted from case reports/case series dealing with diagnostic/therapeutic errors in HC

**Author**	**N. of pts**	**Sample**	**Diagnostic delay**	**Misdiagnoses**	**Treatment received before diagnosis of HC**	**Number/type of physicians consulted prior to correct diagnosis**
Cortijo et al. 2012 [10]	22	Case series selected from a clinical population over a 3-year period	86.1 ± 106.5 mths (range 3–360)	None	NR	NR
Prakash et al. 2010 [14]	4	Case reports	22 yrs, 3 yrs, 2 yrs, 15 mths	Atypical facial pain, atypical odontalgia, sinusitis, caries, pulpitis, psychiatric disorder, chronic migraine	All the patients had dental extractions (6 in one pt), some had sinus surgery, root canal treatment	Several dentists, general physicians, neurologist and ENT specialist (NOS)
Rossi et al. 2009 [15]	25	Case series selected from a clinical population over a 3-year period	5 yrs	Migraine (52%), CH (28%), sinus headache (20%), dental pain (20%), atypical facial pain (16%), stress headache (16%), CEH (8%)	NSAIDs (92%), triptans (32%), antidepressants (32%), and antiepileptics (24%). 36% received invasive treatments. 36% had recourse to complementary and alternative medicine	4.6 (GP 100%, neurologist 80%, ENT specialist 44%, ophthalmologist 40%, dentist 32%, headache specialist 28%)
Taub et al. 2008 [23]	2	Case reports	1.5 yrs; 8 mths	TMD, dental pain, CH, migraine, CPH	Topiramate, nortriptyline, melatonin, verapamil, gabapentin	3 dental practitioners; 1 ENT specialist
Eross et al. 2007 [11]	1	Case report	NR	Sinus headache	NR	NR
Alonso and Nixdorf 2006 [20]	1	Case report	6 mths	Dental pain, CEH	Dental extraction, cervical adjustment, multiple chronic pain medications	4 (dentist, chiropractor, general physician, neurologist)
Benoliel et al. 2002 [21]	1	Case report	2 yrs	Dental pain, migraine, CEH	Dental treatment (NOS), intensive physiotherapy, paracetamol, propranolol, diazepam, ergotamine combination, diclofenac sodium	3 (neurologist, dentist, ENT specialist)

HC: hemicrania continua; CH: cluster headache; CEH: cervicogenic headache; TMD: temporomandibular disorder; CPH: chronic paroxysmal hemicrania; mths: months; yrs: years; NR: not reported; NOS: not otherwise specified.

### Cluster headache

Although recent decades have seen an improvement in the time taken to diagnose CH from onset [8], the diagnostic delay for this condition is still too protracted (more than 3 years in the most recent study [6]), as is the number of physicians consulted before arriving at the correct diagnosis (generally at least 3 medical doctors). A high number of misdiagnoses was described, many of which led to unnecessary invasive and irreversible treatments. CH was most frequently misdiagnosed as: migraine, sinusitis, tooth/jaw problems, and trigeminal neuralgia. **Migraine** seems to be a particularly frequent misdiagnosis. The different temporal patterns of migraine and CH attacks should make it possible to distinguish between these two conditions in the typical case, but if this aspect is not reported by the patient or thoroughly investigated by the physician, confusion may arise, given that many other features of these headaches can overlap. Migraine pain is frequently severe in intensity and unilateral in 2/3 of patients [28]. In about 56% of migraine patients at least one CAS (i.e. lacrimation or conjunctival injection) is present during attacks [29]. Moreover, typical migraine features are often associated with CH attacks. A study of a large cohort of German CH patients found that CH attacks were associated with photophobia or phonophobia in 61.2% and with nausea and vomiting in 27.8%, while migraine aura preceded CH attacks in almost a quarter of the patients [17]. Unfortunately, the ICHD-II fails to mention (either in the diagnostic criteria or in the definitions and comments) that CAS may be present in migraine and that nausea, vomiting, and photo/phonophobia may be present in CH. For clinicians, it is helpful to note that photo- and phonophobia tend to be unilateral in TACs and HC while they are bilateral in migraine [28,30], moreover nausea and vomiting are generally more frequent in migraine than in CH (especially if they occur together) [31]. Another feature that might increase the risk of misdiagnosing CH as migraine is the possibility of the pain switching sides between attacks or cluster periods [18]. Many physicians, even headache specialists, are not aware that this can happen in CH. Indeed, according to the diagnostic criteria for CH (ICHD-II, code 3.1) the pain is unilateral; furthermore, the description paragraph states that it is “strictly unilateral” while the comments section specifies that the “pain almost invariably recurs on the same side during an individual cluster period” [1]. Yet, up to 14% of CH patients may experience a side shift of pain during a cluster period**,** and 18% may have side shifts from one cluster period to the next [32]. A previous diagnosis of **tooth/jaw problems** is likely to be found in the history of CH patients, as 37% to 50% of them reported that the pain radiated to the lower jaw, upper jaw or cheek [6,18,32]. This comes from the fact that patients with CH often describe the pain as emanating from the midfacial region, which might be interpreted as pain originating from the teeth, jaws or temporomandibular joints. However the presence of unilateral attacks associated with relevant ipsilateral CASs that remit spontaneously within 2–3 hours even if untreated, and that relapse with a clock-like periodicity are strong clues for CH. **Sinus headache** (SH) is another misdiagnosis often encountered in clinical practice. According to studies on clinic-based and clinic-based plus non-clinic-based CH populations, this misdiagnosis is made in between 21% [18] and 23% [6] of CH patients. The Sinus, Allergy and Migraine Study - SAMS [11], which, adopting a different perspective, investigated 100 individuals recruited from the general population who believed they had SH, found one who fulfilled the diagnostic criteria for CH. These errors are probably due to the pain localization in CH (frontal region and upper face) and the fact that the picture typically includes CAS referred to the nose, e.g. rhinorrhea/nasal obstruction. However, whereas nasal discharge in SH is thick, purulent, malodorous and frequently accompanied by systemic symptoms such as fever, chills and sweats, in CH it is clear and fluid [1]. Another clinical feature strongly suggesting a diagnosis of CH is a clock-like regularity of attacks. CH patients are also often wrongly diagnosed with **trigeminal neuralgia** (TN). Even though this scenario has been clearly reported by just one study (in which 16% of CH patients had previously been diagnosed with TN), [6] it is a situation that we have frequently encountered in our clinical practice. While the localization of the pain and its duration may, to an extent, be considered somehow similar (although duration it is a matter of seconds in TN versus many minutes in CH), there are many differences between the two conditions that should aid in the differential diagnosis. These include the presence of CAS, the clock-like periodicity of the attacks, and the presence of nocturnal attacks in CH (but not in TN) and the presence of trigger points (only in TN). Nevertheless, TN is, for some reason, the first disorder that many non-headache specialists think of when faced with a patient with a recurrent facial pain condition. Headache specialists should also bear in mind the existence of cluster-tic syndrome, a rare condition characterized by coexistence of CH and TN [33,34].

With regard to treatment, many patients, in the course of the long diagnostic work-up of their CH, were administered inappropriate therapies (quite often invasive and irreversible, i.e. dental procedures and ENT surgery). Moreover, even after the correct diagnosis, many of these patients were still prescribed with treatments not considered first-line options for CH according to the international guidelines [5] (e.g. acute treatments such as opioids or oral triptans or preventive treatment such as propranolol, amitriptyline, carbamazepine and cyproheptadine). We are also aware, from clinical reports at congresses and from our personal observations, of instances in which other non-first-line medications, such as flunarizine or single, high-dose systemic steroid infusion for preventive treatment and indomethacin for acute treatment, were prescribed in CH patients. The above medications have been found to be ineffective in clinical trials [35,36]. Finally, up to 63% of CH sufferers used alternative therapies without finding any of them consistently effective [32].

### Paroxysmal hemicrania

Although our literature review revealed few case series and case reports considering diagnostic pitfalls in PH, this condition appears to be most frequently misdiagnosed as **dental pathologies**. The severe intensity of the pain and its location in the cheek, jaw and maxillary areas in some attacks of PH (that in 1/3 of the cases can be pulsating in quality), may explain this confusion with dental-related pain [22]. However, the short duration of the attacks and the presence of CAS should lead the physician to the correct diagnosis. The localization of PH in the temporal, maxillary and occasionally in the ear regions, along with a certain, ipsilateral masticatory muscle tenderness, can lead to its misdiagnosis as **pain associated with temporomandibular disorder (TMD)**[37]. Yet, a diagnosis of TMD requires the presence of at least one of the following symptoms and signs: pain precipitated by jaw movements and/or chewing of hard /or tough food, reduced range of or irregular jaw opening, and tenderness of the joint capsule(s) of one or both TMJs [1]. Moreover, differences in the intensity of the pain (excruciating in PH versus mild-to-moderate aching pain in TMD) should guide the clinician to the correct diagnosis [37]. The excruciating intensity of PH pain, which also can involve the territories of the second and even third trigeminal branches, and its intermittent temporal pattern may result in an incorrect diagnosis of **trigeminal neuralgia** (TN), especially in the ca. 10% of PH patients in whom attacks can be precipitated by mechanical triggers [38]. However, the triggers in the two conditions differ: in PH, attacks can be precipitated by head flexion or rotation or external pressure over the C2 root, the transverse processes of C4-C5, or the greater occipital nerve on the symptomatic side [38], whereas in TN they can be triggered by actions such as washing the face, shaving, smoking, talking and/or brushing the teeth, or by touching certain small areas in the nasolabial fold and/or chin [1,39]. Unfortunately, the ICHD-II mentions trigger factors only in TN. Had it also mentioned their role in PH, the misdiagnosis rate might be lower. Other features making it possible to differentiate between these two conditions are CAS (present in PH, absent in the majority of TN cases, with exception for TN of the first branch), the duration of the pain (from a few seconds to 2 minutes in TN versus 2–30 minutes in PH), and the nocturnal occurrence of attacks (possible in PH, awakening the patient from sleep**,** but unusual in TN) [22]. The possibility of PH-tic syndrome, similar to cluster-tic syndrome, should also be borne in mind, even though it is a very rare condition [40]. **Cervicogenic headache (CEH)** is a unilateral side-locked headache associated with evidence of cervical involvement (provocation of pain by movement of the neck or by pressure on the neck) [41]. CEH seems to be the most frequently occurring of the hitherto well-known, unilateral headaches. i.e. at 2.2% [42]. Because PH is also a unilateral side-locked headache that can be triggered by neck movement/external pressure in which the pain sometimes involves the neck and occipital areas [38], it can be mistaken for CEH. Although this review did not identify published cases of PH clearly misdiagnosed as CEH, in the authors’ clinical experience this wrong diagnosis can occur. Elements to consider in order to distinguish PH from CEH are: associated CAS (present in PH, absent in CEH), the intensity of the pain (severe or excruciating in PH, moderate in CEH), and its temporal pattern (frequent, short-lasting attacks in PH, versus pain episodes of varying duration or fluctuating continuous pain in CEH). A complete response to indomethacin administration and/or a lack of efficacy of root-nerve blockade further corroborate a diagnosis of PH. Benoliel and Sharav considered the difficulty of differentiating **CH** from PH, given the broad clinical overlap between the two conditions, although they did not report specific cases of misdiagnosis [22]. In this regard, it is helpful to remember some differences between the two conditions such as frequency and duration of attacks (more frequent and shorter in PH than in CH), the sex dominance (male in CH and female in PH) and the patient behavior during the attacks (restless/agitated in CH and generally more quiet in PH). A positive response to indomethacin administration (the Indotest) is a *sine qua non* for the diagnosis of CPH [35]. A properly administered Indotest would prevent not only an incorrect diagnosis, but also the possibility to be prescribed with inappropriate treatment, pharmacological or surgical (multiple tooth extractions, stellate ganglion blocks, cervical sympathetic blocks, trigeminal sensory root section, infraorbital nerve section, sphenopalatine anesthetic injection and gangliectomy, infiltration of the point of Arnold, ethmoidosphenectomy) [25,38].

### SUNCT

We identified only two published cases of SUNCT misdiagnosed as other conditions; in both cases TN was one of the wrongly diagnosed conditions. Differentiating SUNCT from **TN** can be challenging, because the conditions have significantly overlapping clinical phenotypes. The main aspects to take into account include: autonomic features (prevalent in SUNCT and rare in TN), the localization of the pain (V1 in SUNCT and V2/3 in TN), and refractory periods (absent in SUNCT and present in TN) [43]. **Primary stabbing headache** (PSH) is an idiopathic condition, commonly experienced also by people with other primary headaches such as migraine (about 40%) and CH (about 30%). PSH is characterized by unilateral but erratic, moderate-to-severe, jabbing or stabbing pain, lasting from a fraction of a second to 3 seconds [1] or more (in the Vågå study there were also cases of “prolonged jabs” that may last 10–120 sec). PSH can be differentiated from SUNCT on the basis of the site and radiation of the pain (that often varies from one attack to the other), the lack of CAS and triggers [44], and the shorter duration of the attacks (usually less than five seconds, versus a mean of 49 seconds in SUNCT) [45]. SUNCT can also be misdiagnosed as **dental pain**. A review of TACs from the perspective of their implications for dentistry reported cases in which patients with SUNCT, in addition to experiencing facial pain, complained of pain radiating to adjacent teeth [37]. This resulted in therapeutic interventions for dental pain, such as extractions, occlusal splints and incorrect drug treatments. Other therapeutic errors have stemmed from incorrect diagnosis of SUNCT as primary headache syndromes such as TN, atypical migraine, and CH (see Table 3).

### Hemicrania continua

There are several reported cases of HC mimicking **dental pain** or **TMD**. According to a review and case reports on HC, patients can mistake their HC symptoms for toothache or TMD [14,46]. Rossi et al. described 25 patients fulfilling the ICHD-II criteria for HC selected among 1612 subjects attending an Italian Headache Center over a three-year period. Fifty-two percent of these patients had previously been misdiagnosed with **migraine**[15]. This is probably due to the fact that certain migraine features (pain-related ones and associated symptoms) can also occur in HC. Indeed the 40% of the HC patients described by Rossi et al. met the ICHD-II criteria for migraine during HC pain exacerbations [15]. A less common feature of HC that might easily lead to misdiagnosis is the occurrence, reported in four patients, of migraine aura before or during the pain exacerbation [47]. Seven of the 25 HC patients described by Rossi et al. had previously been incorrectly diagnosed with **CH**[15]. According to the authors, this was probably due to the fact that 32% of their HC patients fulfilled the diagnostic criteria for CH during pain exacerbations, and also to the tendency of HC patients to describe only their most severe headache, failing to report the presence of a persistent low-level headache. This might lead to a wrong diagnosis: physicians who investigating a case of episodic head/facial pain syndrome should always seek to establish whether the patient also experiences a lower intensity pain. We identified four cases (reported in three different papers [15,20,21]) of HC patients wrongly diagnosed with **CEH.** Both CEH and HC are side-locked unilateral headaches with a continuous temporal pattern (CEH can have either an episodic or a continuous fluctuating pattern) that can be accompanied by signs and symptoms of neck involvement (always present in CEH, and common in HC too [48]) and by migrainous features [48] (although the degree and the frequency of these associated features is different – i.e. the mean ratio migraine/CEH for the presence of other symptoms were almost 5 for nausea, 4 for throbbing quality of pain, ca 3.5 for photophobia [42]). The response to the Indotest and/or to anesthetic blockade can definitively differentiate between these two similar conditions [35]. **SH** as a misdiagnosis of HC was reported not only by Rossi et al. [15] but also in the Sinus, Allergy and Migraine Study [11]. The most important clinical difference concerns the nasal discharge (clear and fluid in HC but “infectious” in SH). In the routine clinical work-up, nasal endoscopic data, CT and/or MRI imaging and/or laboratory evidence of acute or acute-on-chronic rhinosinusitis are needed, diagnostically [1]. Therapeutic errors in HC are always secondary to misdiagnosis of the condition (as in PH, indomethacin response is a diagnostic criterion of HC) and patients can undergo not only wrong pharmacological treatments, but also unnecessary dental extractions, TMD or ENT surgery, physical therapy, or complementary and alternative medicine therapies [15,20,21,23].

## Discussion and conclusions

In this study, we set out to collect, for the first time, all the original papers referring to diagnostic and therapeutic errors in TACs and HC. Our Medline search strategy detected 13 original articles out of the overall 22 papers that we were able to find in literature (including also the manual search) and that were focused on this topic. These 13 manuscripts included all the major studies conducted in this area; indeed, 2593 of the total of 2614 patients were from studies reported in these 13 manuscripts. On this basis, the Pubmed search strategy that we set up can be deemed satisfactory. On the other hand, we cannot exclude the possibility that our search methods missed some articles not specifically focusing on this topic, but nevertheless containing data of interest for our purposes. Moreover, the likelihood that all data on errors in diagnosis and/or management of these conditions cannot be found in the literature is overwhelming. Not all physicians are inclined to report and/or search and publish “errors”. This is true not just for TACs and HC, but generally for all diseases. Nevertheless we have tried to supplement the relatively scarce literature data by also reporting our own experiences in clinical practice, and the experiences of colleagues (reported to us directly or at conferences).

A novel aspect of this study was the attempt to identify the causes of errors (and the context in which they occurred) in order to understand them better and offer advice on how they might be avoided.

Some of the diagnostic errors identified in this study derived from the fact that non-headache specialists (not only general physician but also ENT surgeons, ophthalmologists and dentists) often are unaware of the less common nosological entities. There is thus a need for specific training in this regard. However, the reported literature also shows that neurologists and headache specialists are liable to making diagnostic mistakes. There may be different reasons for this. The fact that the best diagnostic tool for headache disorders, ICHD-II (well-known and frequently consulted by headache specialists), fails to mention certain clinical features shared by TACs and HC, such as the localization of the pain (which frequently involves the midface, teeth and TMJ, and can switch sides) and associated symptoms (gastrointestinal, photo/phonophobia, aura, etc.) might lead even headache experts to making wrong diagnoses. To reduce the frequency of diagnostic errors, we suggest that these elements should be included in the forthcoming ICHD-III, at least in the comments sections.

The majority of the observed therapeutic errors are due to misdiagnoses. However, even correct diagnoses are no guarantee of an optimal therapeutic approach. For example, drugs not constituting the first-line treatment were reportedly prescribed for correctly diagnosed CH [6], in spite of the availability of updated international therapeutic guidelines for this condition [5].

In conclusion, the results of this review underline the need, alongside the current useful international diagnostic criteria and therapeutic guidelines, for more education concerning TACs and HC, in order to improve their recognition and management.

## Competing interests

The authors declare that they have no competing interests.

## Authors’ contributions

MV and FA designed this review. MV performed the electronic literature search. MV, FA and OS performed the manual search. MV and FA carried out the data extraction. All authors have made substantial contributions to analysis and interpretation of data, have been involved in drafting the manuscript or revising it critically for important intellectual content. All authors read and approved the final manuscript.

## References

[B1] Headache Classification Subcommittee of the International Headache S The international classification of headache disorders: 2nd edition Cephalalgia 2004 14 Suppl 1 9 160 10.1111/j.1468-2982.2003.00824.x 14979299

[B2] Goadsby PJ Cittadini E Cohen AS Trigeminal autonomic cephalalgias: paroxysmal hemicrania, SUNCT/SUNA, and hemicrania continua Semin Neurol 2010 14 2 186 191 20352588 10.1055/s-0030-1249227

[B3] Pareja JA Vincent M Antonaci F Sjaastad O Hemicrania continua: diagnostic criteria and nosologic status Cephalalgia 2001 14 9 874 877 11903280 10.1046/j.1468-2982.2001.00276.x

[B4] Sjaastad O Vincent M Indomethacin responsive headache syndromes: chronic paroxysmal hemicrania and Hemicrania continua. How they were discovered and what we have learned since Funct Neurol 2010 14 1 49 55 20626997

[B5] May A Leone M Afra J Linde M Sandor PS Evers S Goadsby PJ Force ET EFNS guidelines on the treatment of cluster headache and other trigeminal-autonomic cephalalgias Eur J Neurol 2006 14 10 1066 1077 16987158 10.1111/j.1468-1331.2006.01566.x

[B6] Van Alboom E Louis P Van Zandijcke M Crevits L Vakaet A Paemeleire K Diagnostic and therapeutic trajectory of cluster headache patients in Flanders Acta Neurol Belg 2009 14 1 10 17 19402567

[B7] Alore PL Jay WM Macken MP SUNCT syndrome: short-lasting unilateral neuralgiform headache with conjunctival injection and tearing Semin Ophthalmol 2006 14 1 9 13 16517438 10.1080/08820530500509317

[B8] Bahra A Goadsby PJ Diagnostic delays and mis-management in cluster headache Acta Neurol Scand 2004 14 3 175 179 14763953 10.1046/j.1600-0404.2003.00237.x

[B9] Bittar G Graff-Radford SB A retrospective study of patients with cluster headaches Oral Surg Oral Med Oral Pathol 1992 14 5 519 525 1518633 10.1016/0030-4220(92)90088-8

[B10] Cortijo E Guerrero AL Herrero S Mulero P Munoz I Pedraza MI Penas ML Rojo E Campos D Fernandez R Hemicrania continua in a headache clinic: referral source and diagnostic delay in a series of 22 patients J Headache Pain 2012 14 7 567 569 22821619 10.1007/s10194-012-0471-4 PMC3444545

[B11] Eross E Dodick D Eross M The sinus, allergy and migraine study (SAMS) Headache 2007 14 2 213 224 17300361 10.1111/j.1526-4610.2006.00688.x

[B12] Hoffert MJ Headaches that masquerade as dental pain J Mass Dent Soc 1995 14 1 33 35 9520692

[B13] Klapper JA Klapper A Voss T The misdiagnosis of cluster headache: a nonclinic, population-based, Internet survey Headache 2000 14 9 730 735 11091291 10.1046/j.1526-4610.2000.00127.x

[B14] Prakash S Shah ND Chavda BV Unnecessary extractions in patients with hemicrania continua: case reports and implication for dentistry J Orofac Pain 2010 14 4 408 411 21197513

[B15] Rossi P Faroni J Tassorelli C Nappi G Diagnostic delay and suboptimal management in a referral population with hemicrania continua Headache 2009 14 2 227 234 19222596 10.1111/j.1526-4610.2008.01260.x

[B16] Sarlani E Schwartz AH Greenspan JD Grace EG Chronic paroxysmal hemicrania: a case report and review of the literature J Orofac Pain 2003 14 1 74 78 12756934

[B17] Schurks M Kurth T de Jesus J Jonjic M Rosskopf D Diener HC Cluster headache: clinical presentation, lifestyle features, and medical treatment Headache 2006 14 8 1246 1254 16942468 10.1111/j.1526-4610.2006.00534.x

[B18] van Vliet JA Eekers PJ Haan J Ferrari MD Dutch RSG Features involved in the diagnostic delay of cluster headache J Neurol Neurosurg Psychiatry 2003 14 8 1123 1125 12876249 10.1136/jnnp.74.8.1123 PMC1738593

[B19] Sjaastad O Bakketeig LS Cluster headache prevalence. Vaga study of headache epidemiology Cephalalgia 2003 14 7 528 533 12950378 10.1046/j.1468-2982.2003.00585.x

[B20] Alonso AA Nixdorf DR Case series of four different headache types presenting as tooth pain J Endod 2006 14 11 1110 1113 17055919 10.1016/j.joen.2006.02.033

[B21] Benoliel R Robinson S Eliav E Sharav Y Hemicrania continua J Orofac Pain 2002 14 12455433 317 325 12455433

[B22] Benoliel R Sharav Y Paroxysmal hemicrania. Case studies and review of the literature Oral Surg Oral Med Oral Pathol Oral Radiol Endod 1998 14 3 285 292 9540085 10.1016/s1079-2104(98)90010-5

[B23] Taub D Stiles A Tucke AG Hemicrania continua presenting as temporomandibular joint pain Oral Surg Oral Med Oral Pathol Oral Radiol Endod 2008 14 2 e35 e37 18230375 10.1016/j.tripleo.2007.08.043

[B24] Delcanho RE Graff-Radford SB Chronic paroxysmal hemicrania presenting as toothache J Orofac Pain 1993 14 3 300 306 9116630

[B25] Moncada E Graff-Radford SB Benign indomethacin-responsive headaches presenting in the orofacial region: eight case reports J Orofac Pain 1995 14 3 276 284 8995927

[B26] Jensen RM Lyngberg A Jensen RH Burden of cluster headache Cephalalgia 2007 14 6 535 541 17459083 10.1111/j.1468-2982.2007.01330.x

[B27] Benoliel R Sharav Y SUNCT syndrome: case report and literature review Oral Surg Oral Med Oral Pathol Oral Radiol Endod 1998 14 2 158 161 9503449 10.1016/s1079-2104(98)90419-x

[B28] Sjaastad O Cluster headache syndrome 1992 W.B. Saunders, London

[B29] Lai TH Fuh JL Wang SJ Cranial autonomic symptoms in migraine: characteristics and comparison with cluster headache J Neurol Neurosurg Psychiatry 2009 14 10 1116 1119 18931007 10.1136/jnnp.2008.157743

[B30] Irimia P Cittadini E Paemeleire K Cohen AS Goadsby PJ Unilateral photophobia or phonophobia in migraine compared with trigeminal autonomic cephalalgias Cephalalgia 2008 14 6 626 630 18422722 10.1111/j.1468-2982.2008.01565.x

[B31] Ekbom K A clinical comparison of cluster headache and migraine Acta Neurol Scand 1970 14 Suppl 41 1 48 4988867

[B32] Bahra A May A Goadsby PJ Cluster headache: a prospective clinical study with diagnostic implications Neurology 2002 14 3 354 361 11839832 10.1212/wnl.58.3.354

[B33] Green M Apfelbaum R Cluster-tic syndrome headache Headache 1978 14 112

[B34] Solomon S Apfelbaum RI Guglielmo KM The cluster-tic syndrome and its surgical therapy Cephalalgia 1985 14 2 83 89 4016920 10.1046/j.1468-2982.1985.0502083.x

[B35] Antonaci F Costa A Ghirmai S Sances G Sjaastad O Nappi G Parenteral indomethacin (the INDOTEST) in cluster headache Cephalalgia 2003 14 3 193 196 12662186 10.1046/j.1468-2982.2003.00495.x

[B36] Antonaci F Costa A Candeloro E Sjaastad O Nappi G Single high-dose steroid treatment in episodic cluster headache Cephalalgia 2005 14 4 290 295 15773826 10.1111/j.1468-2982.2004.00855.x

[B37] Balasubramaniam R Klasser GD Delcanho R Trigeminal autonomic cephalalgias: a review and implications for dentistry J Am Dent Assoc 2008 14 12 1616 1624 19047667 10.14219/jada.archive.2008.0103

[B38] Antonaci F Sjaastad O Chronic paroxysmal hemicrania (CPH): a review of the clinical manifestations Headache 1989 14 10 648 656 2693408 10.1111/j.1526-4610.1989.hed2910648.x

[B39] Silberstein SD Lipton RB Dodick D Wolff HG Wolff’s headache and other head pain 2008 8 Oxford University Press, Oxford, New York

[B40] Mathew NT Cluster headache and other trigeminal autonomic cephalalgias diagnostic criteria Handb Clin Neurol 2010 14 421 429 20816441 10.1016/S0072-9752(10)97035-8

[B41] Sjaastad O Fredriksen TA Pfaffenrath V Cervicogenic headache: diagnostic criteria Headache 1990 14 11 725 726 2074165 10.1111/j.1526-4610.1990.hed3011725.x

[B42] Sjaastad O Cervicogenic headache: comparison with migraine without aura; vaga study Cephalalgia 2008 14 Suppl 1 18 20 18494988 10.1111/j.1468-2982.2008.01610.x

[B43] Sjaastad O Kruszewski P Trigeminal neuralgia and “SUNCT” syndrome: similarities and differences in the clinical pictures. An overview Funct Neurol 1992 14 2 103 107 1607124

[B44] Pareja JA Sjaastad O Primary stabbing headache Handb Clin Neurol 2010 14 453 457 20816445 10.1016/S0072-9752(10)97039-5

[B45] Pareja JA Shen JM Kruszewski P Caballero V Pamo M Sjaastad O SUNCT syndrome: duration, frequency, and temporal distribution of attacks Headache 1996 14 3 161 165 8984088 10.1046/j.1526-4610.1996.3603161.x

[B46] Peres MF Valenca MM Goncalves AL Misdiagnosis of hemicrania continua Expert Rev Neurother 2009 14 9 1371 1378 19769451 10.1586/ern.09.85

[B47] Peres MF Siow HC Rozen TD Hemicrania continua with aura Cephalalgia 2002 14 3 246 248 12047466 10.1046/j.1468-2982.2002.00325.x

[B48] Peres MF Silberstein SD Nahmias S Shechter AL Youssef I Rozen TD Young WB Hemicrania continua is not that rare Neurology 2001 14 6 948 951 11577748 10.1212/wnl.57.6.948

